# Utilization of Guarana Agroindustrial Waste as a Source of Antimicrobial Compounds for Human Pathogenic Bacterial Removal in Wastewater Sample

**DOI:** 10.3390/biotech15040076

**Published:** 2026-09-03

**Authors:** Erickson Oliveira dos Santos, Cleideane Cunha Costa, Pedro Luis Sosa Gonzáles, Priscila Pauly Ribas, Carla Estefani Batista

**Affiliations:** Research Laboratory for Water Reuse, Samsung Research & Development Institute Brazil, Samsung Eletrônica da Amazônia, Manaus 69075-842, Brazil; cleide.c@samsung.com (C.C.C.); pedro.sosa@samsung.com (P.L.S.G.); priscila.pauly@gmail.com (P.P.R.); ceb.dcl17@uea.edu.br (C.E.B.)

**Keywords:** catechin, guarana, antimicrobial activity, agroindustrial waste, wastewater disinfection, *Paullinia cupana*

## Abstract

The guarana seed peel (GSP), an agroindustrial waste, was characterized for its chemical composition and evaluated for its antimicrobial activity as a potential source of antibacterial compounds for wastewater disinfection using UPLC-QTOF-MS analysis. UPLC-QTOF-MS analysis of GSP, its raw extract (GSPE), and its purified extract (GSPP) identified 36 compounds, including catechin and its oligomers: a B-type procyanidin dimer, pavetannin B6 (trimer), and cinnamtannin A2 (tetramer). Principal component analysis (PCA) separated GSPE from GSPP along PC2, and the purification step concentrated flavonoids—mainly catechin and its oligomers—in GSPP. Antimicrobial activity was assessed against the human pathogens *Escherichia coli* and *Salmonella typhimurium*, causing gastroenteritis and invasive disease, and *Staphylococcus aureus*, causing skin and invasive infections. In real wastewater, GSPE (500 mg L^−1^) reduced total coliforms by up to 81 ± 7% and *E. coli* by up to 74 ± 7% within 180 min. Confocal microscopy with the LIVE/DEAD^®^ BacLight™ kit (Thermo Fhisher, Eugene, OR, USA) confirmed loss of membrane integrity in GSPE-treated *Escherichia coli*, and disc diffusion with GSPP produced inhibition zones of 9–11 mm against *Staphylococcus aureus*, *Escherichia coli* and *Salmonella typhimurium* at 5 mg per disc. These results show that guarana seed peel—currently discarded—is a viable source of catechin-rich antibacterial extracts for wastewater disinfection.

## 1. Introduction

Industrial growth and population expansion have made waste management a pressing environmental problem [1,2]. Agroindustrial waste, however, is a rich source of bioactive compounds [3,4], and as Freitas et al. [5] argue, it no longer needs to be treated as a problem and can instead be seen as an opportunity. Many alternatives are suggested and have been studied for the recovery and reuse of agroindustrial wastes, such as pharmacological applications [6,7,8]; food production [9,10,11]; production of biomaterials [12,13,14]; and as an alternative source of energy generation [15,16]. Agroindustry generates large volumes of residues each year, creating environmental and economic burdens for producers and surrounding communities [8]. Plant- and algae-derived materials are increasingly used in wastewater treatment. Microalgae such as *Chlorella* sp. and *Nitella* sp. remove dissolved solids and nutrients from industrial effluents while generating harvestable biomass, and bioactive compounds from the marine alga *Padina* sp. have been used to synthesize antibacterial silver nanoparticles [17,18,19,20]. These studies establish that waste plant biomass can act simultaneously as a treatment agent and as a source of antimicrobial compounds.

In Amazonas State, the activities of orchards, the production of vegetables and grains, and agroforestry systems stand out. Guarana (*Paullinia cupana*, Sapindaceae) is a native species from the Amazon basin and its seeds have been widely used both as supplements and in energy drinks [21,22]. Brazil is the sole producer of guarana in the world and it is estimated that around 50% of the guarana fruit is discarded during guarana seed processing [8]. The seed has been used in a wide variety of applications such as food supplements and energy drinks, owing to its antitumor activity and gastroprotective, antidepressant, antidiarrheal, analgesic, antipyretic, antimicrobial, antioxidant and immunoprotective properties [23,24,25]. These applications are due to guarana being rich in polyphenols compounds such as tannins (up to 16%); flavonoids, including, catechin and epicatechin; and other substances such as caffeine (2–8%), methylxanthines, theophylline and theobromine, terpenes, coenzyme Q10, benzoic acid, lauric acid, myristic acid, palmitoleic acid, palmitic acid, linoleic acid, oleic acid, stearic acid, and sucrose [23,25,26,27,28,29,30,31].

Another relevant application of these bioactive compounds is in wastewater treatment. In recent studies published in 2004 [26], our research group demonstrated that using guarana (*Paullinia cupana*) agroindustrial waste in coagulation processes achieves a turbidity reduction of over 88% in wastewater treatment, outperforming other materials such as cassava (77%), *pomegranate* (85%), *Stenocereus griseus* (67%), and *Moringa oleifera* (53.7%) in reducing turbidity in domestic wastewater samples’ [27]. In previous work we showed that the organic extract of guarana seed peel acts as a coagulant, removing more than 90% of turbidity and more than 95% of suspended solids from real municipal wastewater at pH 4.0, with sedimentation essentially complete within 15 min [26], outperforming other materials such as cassava (77%), *pomegranate* (85%), *Stenocereus griseus* (67%), and *Moringa oleifera* (53.7%) in reducing turbidity in domestic wastewater samples.

This current study aims to elucidate the antimicrobial activity of agroindustrial waste, demonstrating that this effect is independent of coagulation process. This work was divided into three steps: (i) identifying the compounds present in the peel, its raw extract (GSPE) and its purified extract (GSPP) by UPLC-QTOF-MS; (ii) quantifying the reduction in total coliforms and *E. coli* in real wastewater treated with GSPE, against a pH-matched control without extract; and (iii) testing the antibacterial activity of GSPP against *Staphylococcus aureus*, *Escherichia coli* and *Salmonella typhimurium* by disc diffusion.

## 2. Materials and Methods

### 2.1. Raw Material Stabilization and Preparation of Organic Extracts

Guarana seed peels were used to prepare the extracts tested in the antimicrobial assays. The raw material was donated by an agroindustry from Amazonas State. This agroindustry is located in the Maués municipality, the larger producer of guarana in Amazonas State. The study was registered in National System of Genetic Resource Management and Associated Traditional Knowledge (SisGen AD13ACE).

The peels were dried at 70 °C in a hot-air oven (QUIMIS, Q317M32, São Paulo, Brazil) until constant mass. The dried peels were then milled in a knife mill (SOLAB, SL-31, São Paulo, Brazil) and sieved using a vibratory sieve shaker (SPLABOR, SP-1100, São Paulo, Brazil). The powder obtained from guarana seed peel was used to obtain an organic extract.

The seed peel organic extract used was obtained using the solvents water (H_2_O) and acetone (C_3_H_6_O), in a proportion of 1: 1 *v*/*v*, according to Santos et al. [26]. The extract was stored at −40 °C in an ultra-low-temperature freezer (TERRONI, COLD300, São Paulo, Brazil) for 48 h and then freeze-dried (TERRONI, LS6000, São Paulo, Brazil) until no residual moisture remained.

To purify it, the organic extract powder was reconstituted with deionized water (18.2 MΩ·cm a 25 °C) and acetone in proportion 3:1 *v*/*v*, with a final concentration of 30 g.L^−1^, and was processed by preparative liquid chromatography (Waters, LC Prep Autopurification sytem, Milford, CT, USA) by using an XBridge Prep C18 (Waters, Milford, USA) reverse phase column (19 mm × 50 mm × 5 µm particle size). Mobile phase A was deionized water and mobile phase B was acetonitrile (Sigma-Aldrich, Sellze, Germany). The gradient profile was as follows: 5–95% B (0–5 min—curve 8), 95% B (5–6 min—curve 6), 95–5% B (6–8 min—curve 6), 5% B (8–13 min—curve 6) with a flow of 8 mL.min^−1^, with a photodiode array (PDA) detector scanning 200–300 nm, with peak collection monitored at 200, 275, and 280 nm. The purified fraction was collected between 4.22 and 5.45 min (Supplementary Figures S1 and S2). The collected fractions were transferred to 300 mL glass vials, stored at −40 °C for 48 h, and freeze-dried. The resulting powder, referred to as the purified guarana seed peel extract (GSPP), was used for the antibacterial assays and chemical identification.

### 2.2. Chemical Identification of Powders of Seed Peel, Seed Peel Extract, and Purified Seed Peel

Chromatographic analysis was carried out for the identification and characterization of the powders of the seed peel (GSP), seed peel extract (GSPE), and purified seed peel (GSPP). Each sample was prepared at 20 mg L^−1^ in water:acetonitrile (95:5, *v*/*v*) and analyzed on a Waters Acquity UPLC coupled to a Xevo G2-XS QToF (Waters, Milford, USA) mass spectrometer with an electrospray ionization (ESI) source operating in negative mode (more details information in Supplementary File).

Differences between samples and the main discriminating compounds were determined by chemometric analysis in EZInfo (Umetrics, Umea, Sweden) (linked to Progenesis QI) (Nonlinear, Newcastle upon Tyne, UK), using principal component analysis (PCA) and orthogonal partial least squares discriminant analysis (OPLS-DA).

### 2.3. Antibacterial Activity of Guarana Seed Peel Extract

#### 2.3.1. Antibacterial Activity of Guarana Seed Peel Extract In Vitro

Cell viability was assessed with the LIVE/DEAD^®^ BacLight™ Bacterial Viability Kit (Thermo Fisher, Eugene, OR, USA). This kit includes the SYTO 9—a dye that stains green on a fluorescence microscope in a live bacterial cell—and another dye, propidium iodide (PI) stains red in case of a dead bacterial cell. The method for fluorescence analyses was followed as per the guidelines provided by the viability kit supplier. The images were obtained using a confocal microscope (Leica TCS SP8, Wetzlar, Germany). *Escherichia coli* ATCC 25922 was grown in nutrient broth, and 25 mL of log-phase culture was used in each assay [32]. During the experiment, a part of the samples was incubated with the guarana organic extract and another part was not (positive control). Apart from that, a part of the samples was treated with a standard antibiotic, amoxicillin, and with the solvents used in the extraction (negative control). All tests were made in triplicate.

#### 2.3.2. Antibacterial Tests for *E. coli* in Real Sample of Wastewater with Guarana Seed Peel Extract

The guarana extract at a concentration of 500 mg L^−1^ was placed within the jar test equipment, with a real wastewater sample from a wastewater treatment plant, adjusted to pH 4.0 with HCl. The jar test mixing conditions, guarana extract concentration and wastewater sample in pH 4 were determined in previous research, published in 2024 [26]. A control test was also performed in the same condition, where no extract was added in the jar test—this was to verify the natural count of the microorganisms without extract. Samples of 100 mL were collected at eight different time intervals (t = 0 min, 15 min, 30 min, 60 min, 90 min, 120 min, 150 min and 180 min). The test was performed in triplicate.

The detection of coliforms and *E. coli* was made using the Colilert^®^-18 test (Quanti-Tray/2000^®^, Westbrook, ME, USA), performed according to the manufacturer’s instructions. After incubation, large and small wells are counted according to the change in colouring and fluorescence emission (365 nm). Yellow wells are considered positive for total coliform while the wells that emit fluorescence are considered positive for the presence of *E. coli*. The combination of the number of large wells and small positive wells is used to determine the most probable number (MPN). The result of this analysis was expressed as MPN per 100 mL, with confidence greater than 95% [33].

#### 2.3.3. Disc Diffusion Assay with the Purified Seed Peel Extract (GSPP)

The antibacterial effect of the purified organic extract of guarana seed peel (GSPP) was assessed by the disc diffusion method. Three pathogenic bacteria found in water and food were chosen: *Staphylococcus aureus* ATCC^®^ 25923, *Escherichia coli* ATCC^®^ 25922™ and *Salmonella typhimurium* ATCC^®^ 14028™. During the tests, the antibiotic Streptomycin S 300 µg was used as a reference control for antibacterial efficiency. For *S. aureus*, the agar used was mannitol salt in a proportion of 55.51 g in 500 mL; for *E. coli* and *S. typhimurium*, EMB Agar was used in a proportion of 18 g in 500 mL. Media were prepared in Milli-Q water and dissolved in a microwave. They were then transferred to 1 L Duran bottles, sealed, and autoclaved at 121 °C and 1 atm for 20 min. Approximately 20 mL was poured into sterile Petri dishes and left to solidify.

For the assay, a stock solution of GSPP was prepared by weighing 0.400 g of purified extract and dissolving it in 1500 µL of Milli-Q water and 500 µL of acetone, giving a final concentration of 200 mg mL^−1^ (stock solution, SS). Discs were loaded with the SS, corresponding of 80 µg until 5000 µg of GSPP per disc, respectively. The blank was prepared with the same solvent mixture without extract (1500 µL of Milli-Q water and 500 µL of acetone).

Once the bacteria, culture media and extract solutions were ready, discs were loaded with either the blank solvent or the GSPP solution at the defined concentration, and the solvent was allowed to evaporate. Bacteria were inoculated onto fresh Petri dishes, and three discs were then placed on each plate: a blank disc, a disc loaded with GSPP, and a disc loaded with the antibiotic. Plates were incubated at 37 °C for 24 h in a BOD incubator. After this period, the diameter of the inhibition zone formed around each disc was measured.

### 2.4. Artificial Intelligence Statement

An AI-assisted language tool (Claude, Anthropic, 548 Market Street, PMB 90375, San Francisco, CA 94104, USA) was used to revise the English of the manuscript for clarity, grammar, and consistency of terminology and units, and to identify internal inconsistencies between the text, tables, and figures. The tool was not used to generate, analyze, or interpret experimental data, nor to produce figures or draw scientific conclusions. All suggestions were assessed individually by the authors, who verified every numerical value against the original laboratory records and approved the final wording. The authors take full responsibility for the content of the manuscript.

## 3. Results and Discussion

### 3.1. Chemical Identification of Guarana Seed Peel and Its Extracts

UPLC-QTOF-MS analysis of seed peel (GSP) and organic extracts of guarana seed peel extract (GSPE), and seed peel purified (GSPP) allowed the identification of possible chemical markers present in analyzed guarana parts.

Samples were aligned by *m*/*z* and retention time in Progenesis QI. Of the 3151 features detected, 1559 were assigned a putative identity, and 36 met all identification criteria. The dominant signals in GSPE and GSPP were two isomers at *m*/*z* 289.07 (C_15_H_14_O_6_; M-H) followed by *m*/*z* 575, 577, 863 and 1151 (Table S1).

The main *m*/*z* have been identified were previously reported in articles that analyzed guarana seed [2,11,12], but had not been reported in the seed peel. The compound at *m*/*z* 289.0718 ([M−H]^−^) was identified as catechin. The ions at *m*/*z* 577.1348 and 575.1189 correspond to B-type procyanidin dimers, *m*/*z* 863 to pavetannin B6 (trimer), and *m*/*z* 1153.2598 to cinnamtannin A2 (tetramer). Because B-type procyanidin isomers are isobaric, the dimer is reported at the class level; assignment to B1 or B2 would require authentic standards.

**Figure 1:**
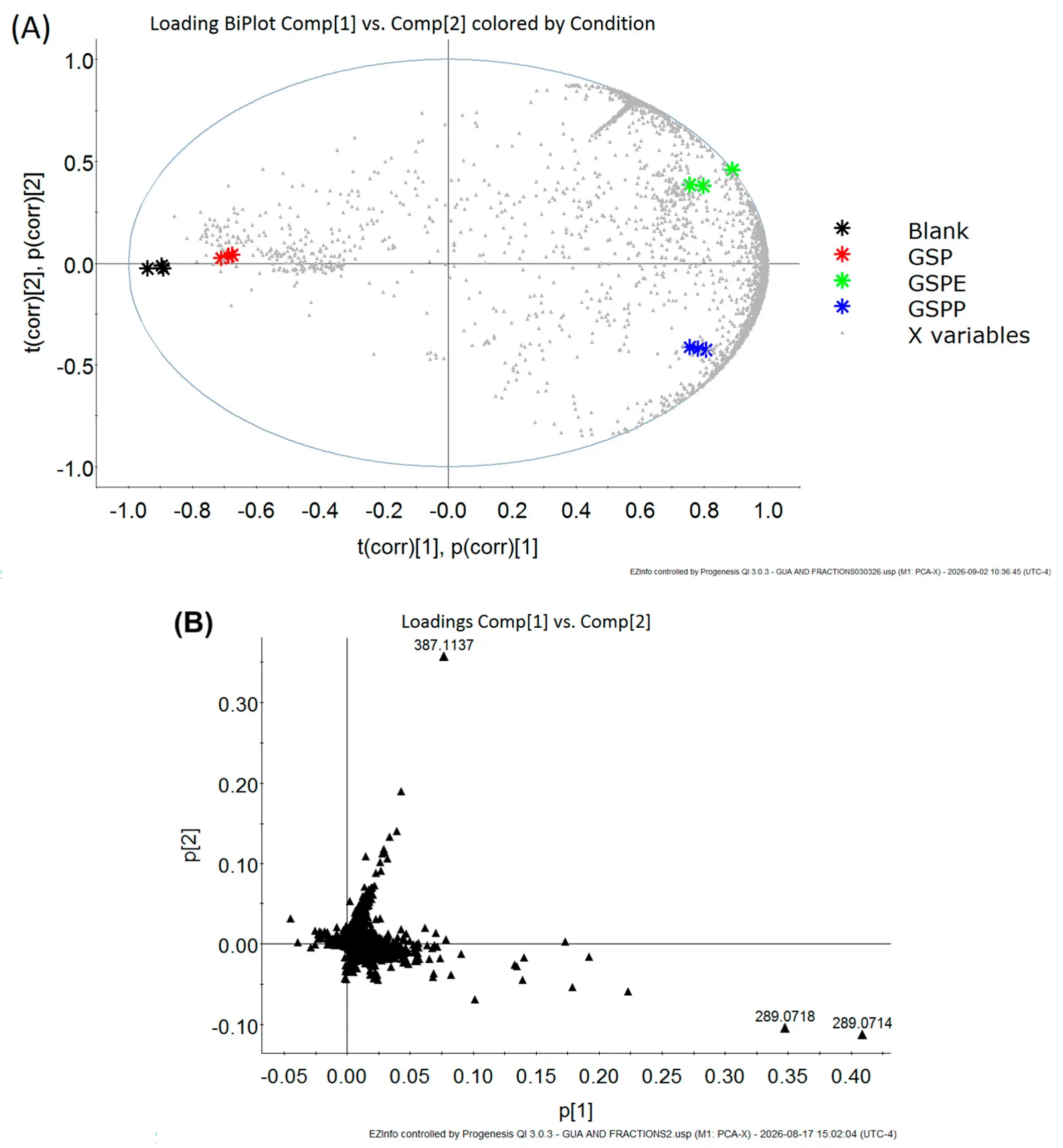
Loading biplot PCA from guarana seed peel (GSP), guarana seed peel extract (GSPE), guarana seed peel purified (GSPP). (**A**) biplot with PC1 and PC2 explain 78.15% of the total cumulative variance. (**B**) PCA Loading of PC1 × PC2 with mass-to-charge ratio (*m*/*z*).

Although GSPE and GSPP showed similar chromatographic profiles and shared the same major ions, PCA separated them: both clustered together along PC1 but fell into opposite quadrants along PC2. Together, PC1 and PC2 explained 78.15% of the total variance (Figure 1A). PC1 accounted for 63.53% and was driven by the catechin isomers at *m*/*z* 289.07, while PC2 accounted for 14.62% and was driven by *m*/*z* 387.1137 and 341.1082, 341.1082 was identified as sucrose, and compound 387.1137 did not meet all identification criteria, but analysis based on the METLIN database suggests that the compound is a 6(5H)-Phenanthridinone (C_13_H_8_NO), with a mass error of −0.62 ppm, a score of 39.3 and an isotope similarity of 97.26 (Figure 1B).

PC1 had loadings with the characteristic masses of flavonoids, especially catechin ([M−H]^−^, *m*/*z* 289.0718) and its dimer and trimer, namely a B-type procyanidin dimer ([2M−H]^−^, *m*/*z* 577.1348) and pavetannin B6 ([3M−H]^−^, *m*/*z* 863.1834).

**Figure 2:**
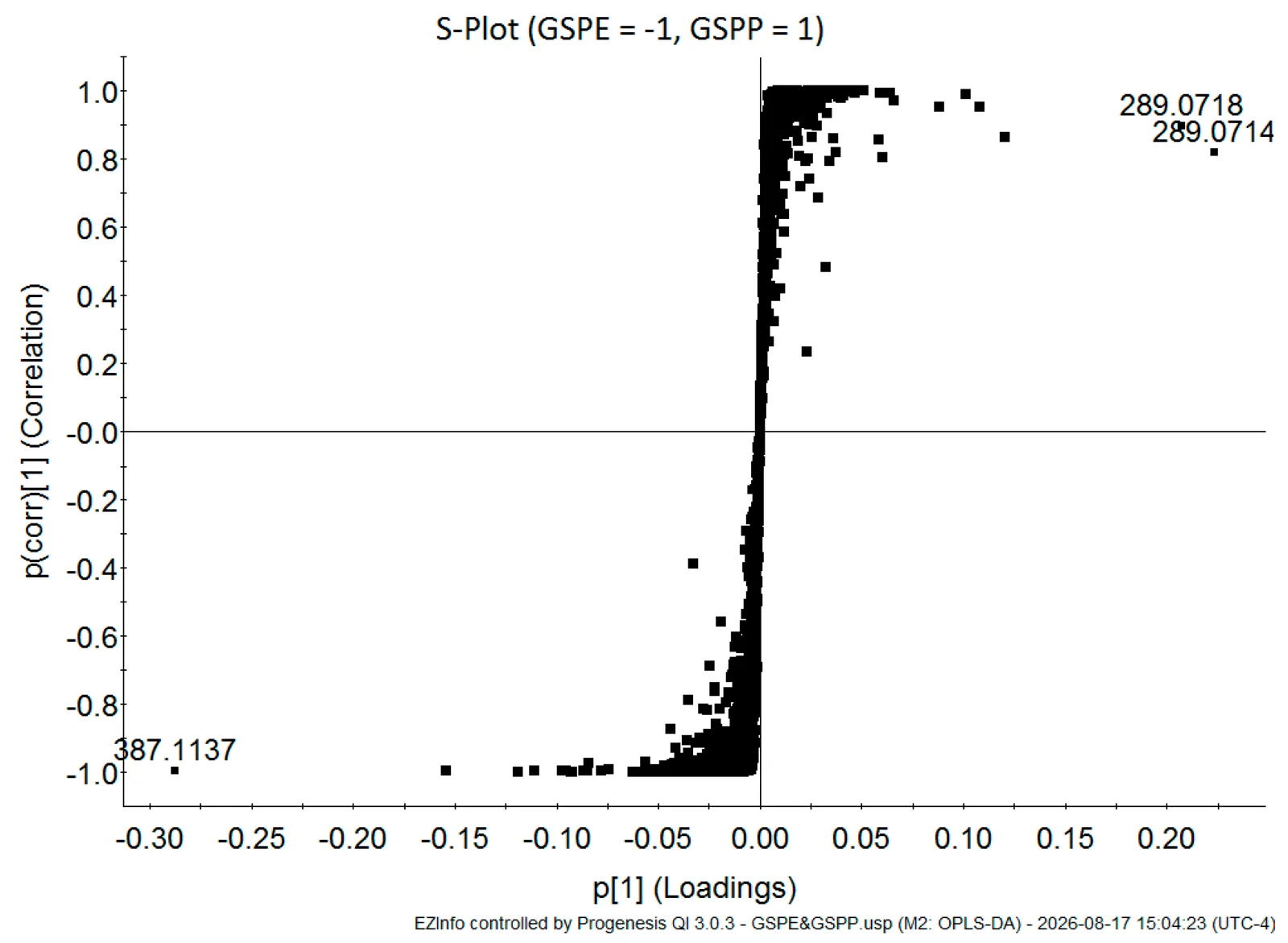
S-plot derived from the OPLS-DA model indicating flavonoids compound concentrated in GSPP (upper right).

The OPLS-DA S-plot separated GSPE from GSPP (Figure 2) model demonstrated exceptional robustness and predictive power, yielding an R^2^Y(Cum) of 99% and a Q^2^(Cum) of 99%. In the S-plot contribution quadrants, a cluster of ions at *m*/*z* 290.0791 (*p* = 0.2069; *p*(corr) = 0.897) and *m*/*z* 290.0787 (*p* = 0.2232; *p*(corr) = 0.8177) stood out as the primary biomarkers associated with GSPP, showing strong positive correlations and high covariance magnitudes, which suggest they represent the same highly abundant molecular entity. Conversely, the ion at *m*/*z* 387 characterized the chemical signature of GSPE, revealing a near-perfect negative correlation (*p*(corr) = −0.997) and substantial covariance (*p* = −0.28).

Consequently, these findings highlight the most prominent molecular divergences between the studied extraction conditions; given that GSPP exhibits targeted antimicrobial activity, these highly distant outliers on the S-plot emerge as the key bioactive candidates driving this biological effect.

### 3.2. Antibacterial Tests in Real Sample of Wastewater with Guarana Seed Peel Extract (GSPE)

**Figure 3:**
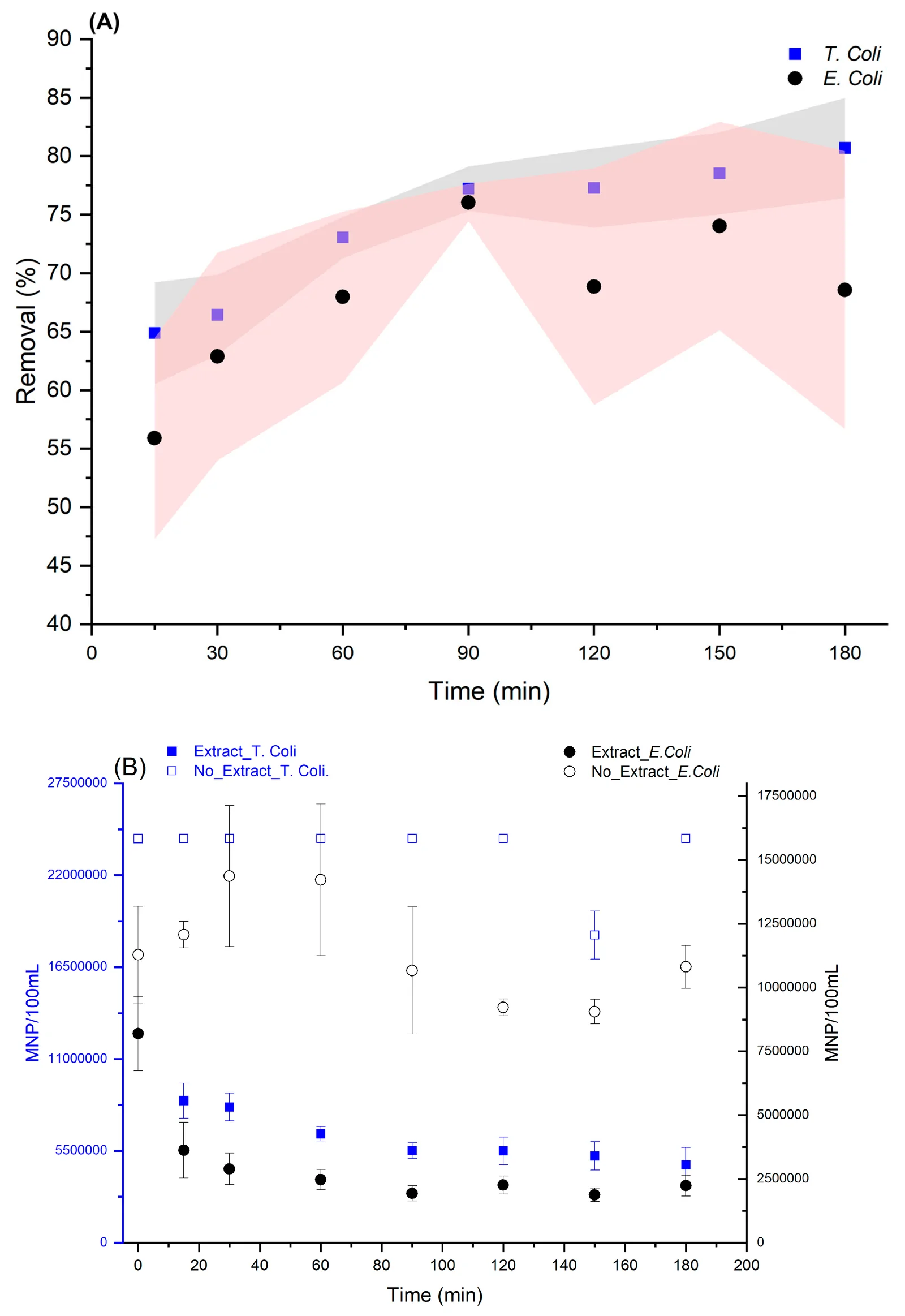
(**A**) Efficiency of removal (%) of total coliforms and *E. coli* in wastewater samples with guarana agroindustrial waste extract. Total coliforms, blue box: mean, and SE of the mean in the grey zone. *E. coli*, black dot: mean, and red zone for SE of mean. (**B**) Jar-test results with MPN (Most Probable Number) data for *E*. *coli* and Total Coli growth; tests conducted with and without extract. Total coliforms, blue box: mean, and *E. coli*, black dot: mean.

The counting of total coliforms and *E. coli* using the Colilert^®^-18 method showed that the microbial concentration in samples treated was lower when compared to the untreated effluent. When analyzed the total coliform counting, it is possible to observe that the maximum reduction occurs after 180 min with 81 ± 7% (mean ± standard deviation; range 73–88%). Inhibition was already evident within 15 min, when a 65 ± 8% (range 57–72%) reduction in the total coliforms counting was observed. The result obtained with GSPE in this study was similar to what was found in previous studies for *Moringa oleifera*. For comparison, *Moringa oleifera* has been reported to reduce total coliforms by 77–91% [34,35]. A similar behaviour was observed in *E. coli* counts after treatment with GSPE. *E. coli* counts fell by 56 ± 15% (45–73%) within 15 min and reached a maximum reduction of 74 ± 7% (56–86%) at 150 min (Figure 3A,B). The efficiency was lower than the reduction obtained with *Moringa oleifera* in Kwabena Ntibrey et al. [35] study, where the reduction in *E. coli* count was >99%.

The effect of organic extracts of guarana seed against *E. coli* was observed by Majhenič et al. [36]; they presented inhibitory activities in *B. cereus*, *P. fluorescens* and spoilage fungi such as *A. niger*, *T. viride* and *P. cyclopium*. We attribute this effect to the antioxidant activity of guarana seeds that result in an inhibitory activity against both Gram-negative and Gram-positive bacteria. The difference between the results found in this article and the one previously published was the raw material; in previous research the guarana seed was the sample, and this research used guarana agroindustrial waste. Guarana seeds have been used for centuries and their medicinal properties are already known [37], while the use of agroindustrial waste is still sparsely studied, showing the relevance of the results obtained.

### 3.3. Antibacterial Tests In Vitro with Seed Peel Extract Purified (GSPP)

**Figure 4:**
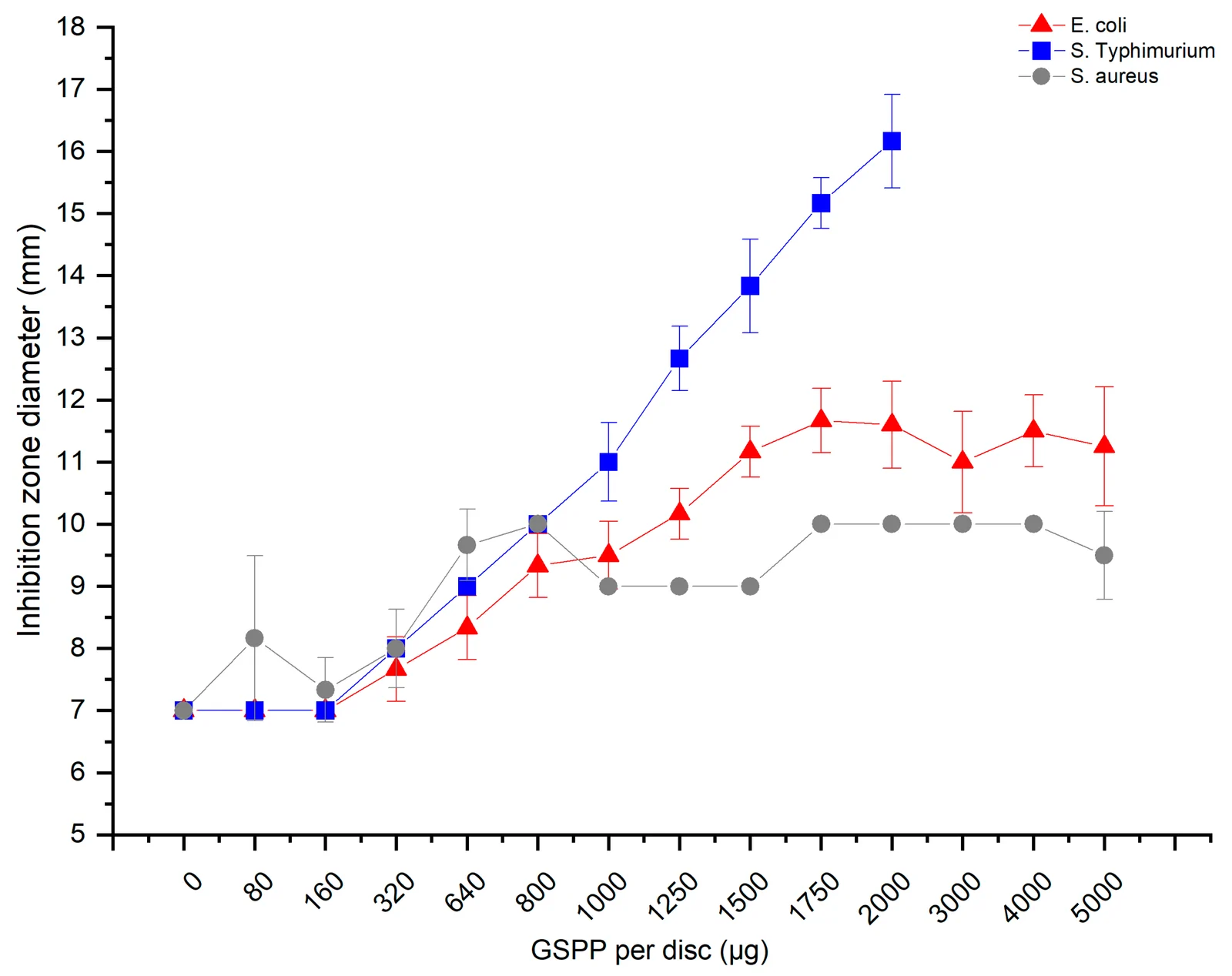
Line graph with standard deviation showing the antimicrobial effect of GSPP—determined via disc diffusion assay across a concentration range of 80 to 5000 µg —against *E. coli*, *S. typhimurium*, and *S. aureus*.

The antibacterial activity of GSPP was evaluated by the disc-diffusion method at 14 concentrations ranging from 80 to 5000 µg per disc. In total, 86 discs were tested against *Escherichia coli*, 52 against *Salmonella typhimurium*, and 39 against *Staphylococcus aureus*. Differences in mean inhibition-zone diameters were analyzed by one-way analysis of variance (ANOVA), followed by Dunnett’s multiple-comparison test, using the blank disc as the control. The greatest inhibitory effect against *S. aureus* was observed at 800 µg per disc, which produced an inhibition zone of 10.0 mm (*n* = 3). In contrast, the maximum inhibition of *S. typhimurium* and *E. coli* was observed at 2000 µg per disc, yielding inhibition zones of 16 ± 0.7 mm (*n* = 6) and 12 ± 0.7 mm (*n* = 10), respectively. These findings demonstrate that GSPP exhibits antibacterial activity against both Gram-positive and Gram-negative bacterial species, although the concentration associated with maximal inhibition differed among the tested organisms (Figure 4).

According to chemical identification and chemometric analysis, GSPP is rich in catechin and its oligomers. Ma et al. [38] reviewed the antibacterial activity of catechins from different plant sources against *Escherichia coli* and *Salmonella*, and the inhibition zones compiled from that work are summarized in Table 1. For *E. coli*, reported zones range from 7 mm for *Libidibia ferrea* [39] to 30 mm for pomegranate [40], with intermediate values for *Parapiptadenia rigida* (8.33 ± 0.58 mm) [41] and common herbs (7.5–11.5 mm) [42]. For *Salmonella*, a zone of 8.33 ± 0.58 mm has been reported for *L. ferrea* [39]. The zones obtained here with GSPP—12 ± 0.7 mm (*n* = 10) mm for *E. coli* and 16 ± 0.7 mm (*n* = 6) for *Salmonella typhimurium*—fall within the lower part of this range, which is expected for a multicomponent extract that has not been fractionated to a single compound. Notably, all of the sources compiled in Table 1 are primary raw materials, whereas the catechins reported here were recovered from a waste stream—the main novelty of this study.

Catechin’s antimicrobial mechanisms are due to its antioxidant properties and ability to interfere with the cell membranes of microorganisms, inhibiting their growth. Catechin modulates the metabolic processes essential for the survival of bacteria and fungi, interfering with the enzymes involved in metabolic reactions, destabilizing cell membranes and affecting the production of proteins essential for bacterial growth. These actions result in bacterial growth inhibition and virulence reduction; catechin’s antioxidant capacity can neutralize reactive oxygen species, reducing oxidative stress in bacterial cells. All these mechanisms combined contribute to catechin’s antimicrobial properties.

## 4. Conclusions

The present study highlights the significant potential of guarana agroindustrial waste, specifically the seed peel, as a valuable raw material for the development of antibacterial products. Through comprehensive chemical identification and UPLC-QTOF-MS analysis, we identified key chemical markers in guarana seed peel (GSP), guarana seed peel extract (GSPE), and guarana seed peel purified (GSPP). Notably, the presence of catechins and their derivatives, including dimers, trimers, and tetramers, was confirmed, with these compounds being previously unreported in guarana seed peel. The chemometric analysis, including PCA and OPLS-DA, revealed distinct profiles between GSPE and GSPP, emphasizing the enrichment of flavonoids in the purified extract.

The antibacterial efficacy of GSPE and GSPP was evaluated in both real wastewater samples and in vitro conditions. GSPE demonstrated efficacy in reducing *E. coli*; however, when the disc diffusion test was conducted, no inhibition was detected. Therefore, purification was carried out in several fractions, and one fraction (GSPP) showed antibacterial activity against *Staphylococcus aureus*, *Escherichia coli*, and *Salmonella typhimurium*. These findings underscore the multitarget nature of catechins and their derivatives in combating bacterial infections, as they disrupt cell membrane integrity, inhibit key metabolic enzymes, and interfere with bacterial communication systems.

The study also emphasizes the environmental and economic benefits of utilizing guarana agroindustrial waste, which is currently discarded without proper disposal. By transforming this waste into a high-value antibacterial product, we contribute to sustainable practices and the circular economy.

## Figures and Tables

**Table 1:** Antibacterial ability of catechins from different sources against *Escherichia coli* and *Salmonella*.

Source	Bacterial	Test Method	Antibacterial Ability	Reference
Pomegranate	*Escherichia coli*	Inhibition zone	30 mm	[40]
Common herbs	*Escherichia coli*	Inhibition zone	7.5–11.5 mm	[42]
*Libidibia ferrea*	*Escherichia coli*	Inhibition zone	7 mm	[39]
*Parapiptadenia rigida*	*Escherichia coli*	Inhibition zone	8.33 ± 0.58 mm	[41]
Guarana Seed peel purified	*Escherichia coli*	Inhibition zone	12 ± 0.7 mm	This study
*Libidibia ferrea*	*Salmonella typhimurium*	Inhibition zone	8.33 ± 0.58 mm	[39]
Guarana Seed Peel purified	*Salmonella typhimurium*	Inhibition zone	16 ± 0.7 mm	This study

## Data Availability

The original contributions presented in this study are included in the article/Supplementary Materials. Further inquiries can be directed to the corresponding author.

## Supplementary Materials

The following supporting information can be downloaded at: https://www.mdpi.com/article/10.3390/biotech15040076/s1, Figure S1: Process for obtaining purified guarana seed extract; Figure S2: Chromatogram of organic extract of guarana agroindustrial waste; Table S1: Chemical identification of guarana seed peel purified extract (GSPP) and guarana seed peel extract (GSPE) by UPLC-QToF-MS; Figure S3: Imaging of a dry sludge sample at a critical point by scanning electron microscopy (SEM); Figure S4: Imaging of the bacterial viability test in the samples treated with guarana agroindustrial waste extract using confocal microscopy; Figure S5: Disc diffusion assay with the purified guarana seed peel extract (GSPP) after incubation for 24 h at 37 °C [43,44,45,46,47,48].
