# Supplementary material for: Utilization of Guarana Agroindustrial Waste as a Source of Antimicrobial Compounds for Human Pathogenic Bacterial Removal in Wastewater Sample
Source: BioTech (Basel). 2026 Sep 3;15(4):76. doi: 10.3390/biotech15040076 (PMC13600032; doi:10.3390/biotech15040076)
Supplement: Supplementary file 1 [file biotech-15-00076-s001.zip › biotech-4444970-supplementary.pdf]

Supplementary Materials: Utilization of guarana Agroindustrial Waste as a source of antimicrobial compounds for human pathogenic bacterial removal in wastewater sample

Erickson Oliveira dos Santos, Cleideane Cunha Costa, Pedro Luis Sosa Gonzáles, Priscila Pauly Ribas and Carla Estefani Batista.

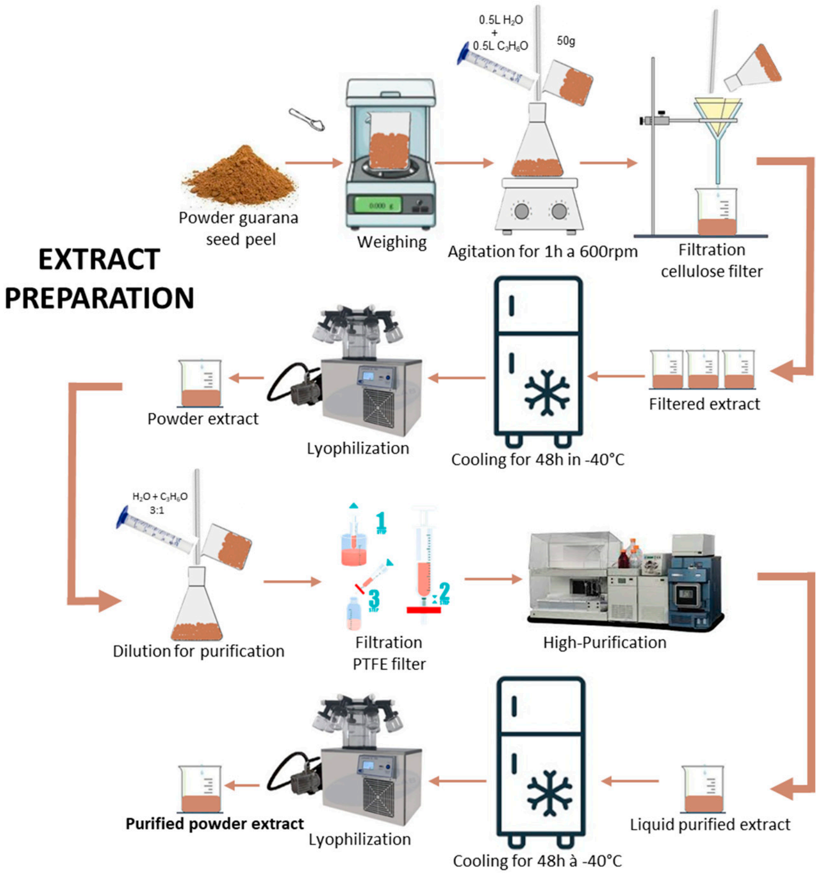

Figure S1. Process for obtaining purified guarana seed extract. Ground powder, extraction 1:1 - H2O:C3H6O v/v, filtration with qualitative cellulose filter, freezing, lyophilization, extraction 3:1 - H2O:C3H6O v/v, filtration with polytetrafluoretileno 0,45µm filter (PTFE), purification, freezing, lyophilization, pure powder obtained.

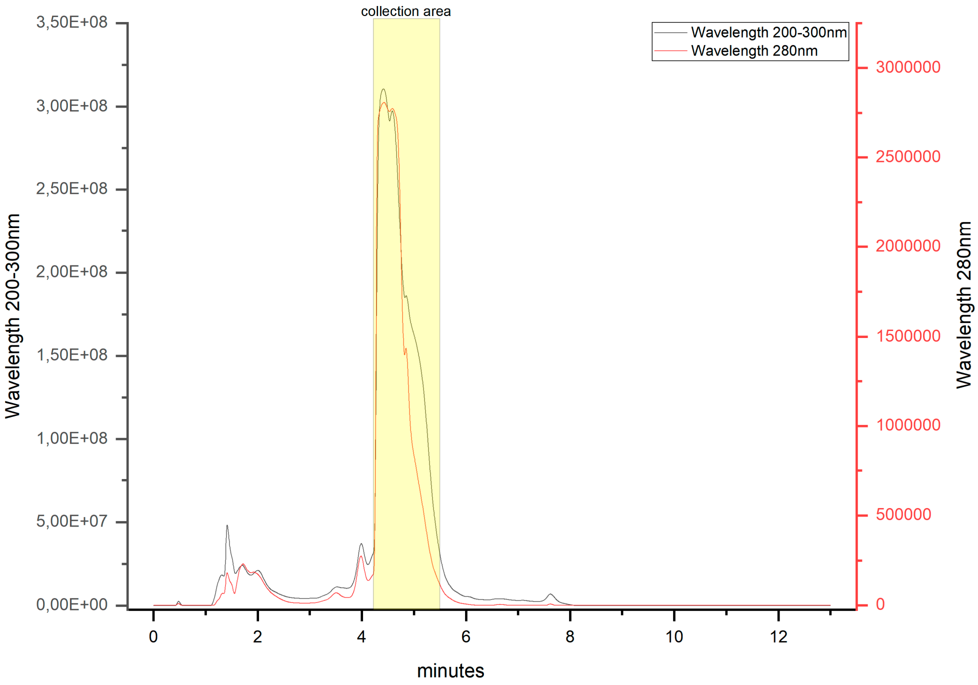

Figure S2. Chromatogram of organic extract of guarana agroindustrial waste. Collected area of purified organic extract is in highlight yellow.

1. Chemical identification of powders of seed peel, seed peel extract, and purified seed peel.

The separation was performed on an Acquity UPLC BEH C18 with reverse phase column (100 mm x 2.1 mm x 1.7 µm particle size). Mobile phase A was 0.01% formic acid in deionized water and mobile phase B was 0.1% formic acid in acetonitrile. The gradient profile was as follows: 5-95% B (0-10 min - curve 8), 95% B (10-11 min - curve 6), 95%-5% B (11-12 min - curve 6), 5% B (12-15 min - curve 6). The injection volume of each sample and standard were 10 µL with flow rate at 0.5 mL/min. The mass spectrometric parameters were as follows: Capillary voltage 3.0kV; cone voltage 40 V; source temperature 100ºC; cone gas flow (N2) 50 l/h; desolvatation gas flow (N_2) 900 l/h; desolvatation temperature 600ºC. Both low (6 eV) and high (ramped from 20 to 35 eV) collision energy data were recorded by employing MS^E continuum mode, acquisition time 0 to 8 minutes, acquisition mode, negative polarity, resolution mode, and extended dynamic range. Data were acquired from 100 to 1500 amu and using a scan time of 0.2s. Leucine enkephalin (m/z 554.2615 $[M-H]^-$) was used as the lock mass calibrant. The accurate mass data of molecular ions were processed using Progenesis QI (nonlinear-Waters). Progenesis was configurated with automatic review alignment; experiment design setup with 4 groups (blank, guarana peel, seed peel and seed); peak picking configurated for 1 and 8 minutes; automatic review decovulation; identification of compounds was carried out with a personal library with 47 compounds found in guarana—METLIN™ MS/MS 2019; in the review, compounds were setup with filters: blank and noise, the blank setup were all m/z with a high abundance in blank samples; the noise setup were all m/z with standard deviation >30 and abundance < 100. The widely accepted accuracy threshold for the confirmation of elemental compositions was established at <5 ppm, an isotopic similarity score >75, and with fragmentation score>0

Table S1. Chemical identification of guarana seed peel purified extract (GSPP) and guarana seed peel extract (GSPE) by UPLC-QToF-MS.

**Highest Mean Compound in GSPP**							
Compound	m/z	Adduct (Rel. abundance)	Fragmentation score	Isotope similarity	Mass erro (ppm)	Max Abundance	R.T (min)
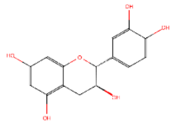	289.0714	M-H (68%)<br>2M-H (29%)<br>3M-H (3%)	52	95.41	-1.14	216,8630	4.65
Catechin ($C_{15}H_{14}O_6$)	289.0718	M-H (76%)<br>2M-H(23%)<br>3M-H(1%)	36.5	96.80	0,17	156,1570	3.10
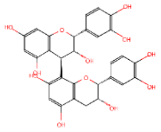<br>Procyanidin B1 ($C_{30}H_{26}O_{12}$)	577.1348	M-H(98%)<br>2M-H(2%)	43.9	91.58	-0.57	44,366	4.24
	863.1834	M-H	69.7	79.44	0.58	40,495	5.67
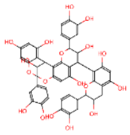<br>Pavetannin B6 ($C_{45}H_{36}O_{18}$)	863.1824	M-H	69.5	96.39	-0.52	22,792	4.72
	577.1344<br>577.134	M-H	55.2<br>62.6	88.17<br>89.13	-1.23<br>-2.03	24,780<br>23,915	5.98<br>3.86

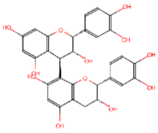 Procyanidin B2 ($C_{30}H_{26}O_{12}$)	577.1349		37.7	87.79	-0.47	10,049	4.54
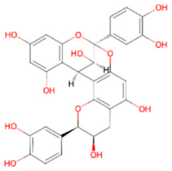 Proanthocyanidin A2 ($C_{30}H_{24}O_{12}$)	575.1199	M-H	55.1	91.20	0.66	14,779	6.52
	575.1189		64.9	78.30	-1.03	9,262	5.63
	575.1195		59.5	90.26	-0.06	6,598	6.06
	575.1203		43	80.14	1.36	1,791	6.90
	1151.2469	2M-H	23.4	80.30	0.57	1,706	5.20
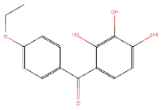 2,3,4-Trihydroxy-4'ethoxybenzophenone ($C_{15}H_{14}O_5$)	273.0761	M-H	20.5	95.45	-2.87	526	5.43
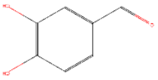 3,4-Dihydroxybenzaldehyde ($C_7H_6O_3$)	137.0243	M-H	50.7	96.65	-1.08	397	2.31
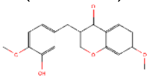 Deoxysappanone B 7,3'Dimethyl Ether ($C_{18}H_{18}O_5$)	313.1084	M-H	0.939	96.45	0.72	387	6.42
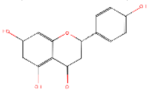 (-)-Naringenin ($C_{15}H_{12}O_5$)	271.061	M-H	19.6	93.86	-0.62	213	4.66
	271.0613		18.9	92.18	0.51	169	3.09
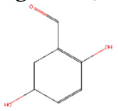 2,5-Dihydroxybenzaldehyde ($C_7H_6O_3$)	137.024	M-H	28.8	96.50	-2.71	161	4.66
	137.0242		28.9	94.09	-1.28	116	3.10
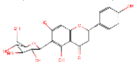 Hemiphloin ($C_{21}H_{22}O_{10}$)	433.1131	M-H	1.59	91.95	-2.10	119	6.50
**Highest mean compound in GSPE**							
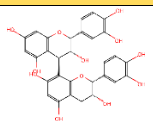 Procyanidin B2 ($C_{30}H_{26}O_{12}$)	577.1362	M-H	64.8	89.91	1.84	36,157	2.65
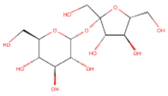 Sucrose ($C_{12}H_{22}O_{11}$)	341.1082	M-H (80%) 2M-H (17%) 3M-H (3%)	16.5	97.13	-2.27	9,324	0.48
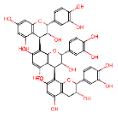	865.1980	M-H	79.2	85.25	-0.58	7,505	3.60
	865.1982		33.4	85.74	-0.42	3,337	4.21
	865.1988		49	82.14	0.30	3,911	4.51

Procyanidin C1 ($C_{45}H_{38}O_{18}$)							
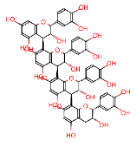 Cinnamtannin A2 ($C_{60}H_{50}O_{24}$)	1153.2598	M-H	63.7	78.26	-1.83	5,738	5.34
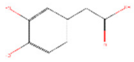 Dihidroxyphenylacetic acid ($C_8H_8O_4$)	168.0421	M-H (1%)<br>2M-H (99%)	33	93.50	-1.18	2,937	4.65
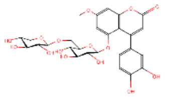 5,3',4'-Trihydroxy-7-methoxy-4-phenylcoumarin 5-O-xylosyl-(1->6)-glucoside ($C_{27}H_{30}O_{15}$)	593.1590	M-H	7.23	96.63	-1.99	1,914	5.11
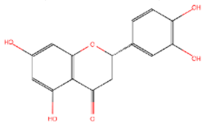 Eriodictyol ($C_{15}H_{12}O_6$)	287.0557	M-H	3.84	97.45	-1.30	940	4.66
	287.0561		3.3	96.69	-0.06	762	3.10
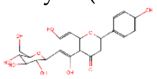 Hemiphloin ($C_{21}H_{22}O_{10}$)	433.1126	M-H	9.91	94.06	-3.31	601	3.73
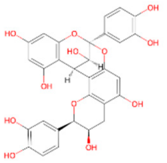 Proanthocyanidin A2 ($C_{30}H_{24}O_{12}$)	1151.2492	2M-H	13.2	80.99	2.53	637	2.28
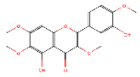 Casticin ($C_{19}H_{18}O_8$)	373.0879	M-H	0.126	91.52	-1.43	134	5.84
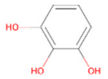 Pyrogallol ($C_6H_6O_3$)	377.0879	3M-H	0.07	95.49	0.30	131	4.98
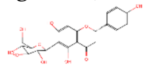 Hemiphloin ($C_{21}H_{22}O_{10}$)	433.1132	M-H	0.186	77.72	(-1.83)	131	2.31

2. Antimicrobial activity via scanning electron and confocal microscopy imaging

SEM images showed fewer bacterial cells in sludge from GSPE-treated effluent than in sludge from untreated effluent (Figure S3.A). These results indicate that guarana agroindustrial waste is efficient in total coliforms and E. coli removal. Vega Andrade et al. [43] showed that the tertiary treatment with both natural and synthetic coagulants resulted in the efficient removal of those microorganisms. These authors explain that the process could be due to the capture of microorganisms by coagulation, flocculation, and subsequent removal by sedimentation. The hypothesis is also developed by Yimer & Dame [44], who concluded that the removal of total coliforms was directly proportional to concentration of colloidal in suspension, which could be possible because of the loss of their natural supporting structure [45]. In our system, sludge formed during treatment with GSPE contained fewer bacteria (Figure S3.B), which is consistent with removal by coagulation and sedimentation rather than with cell lysis alone.

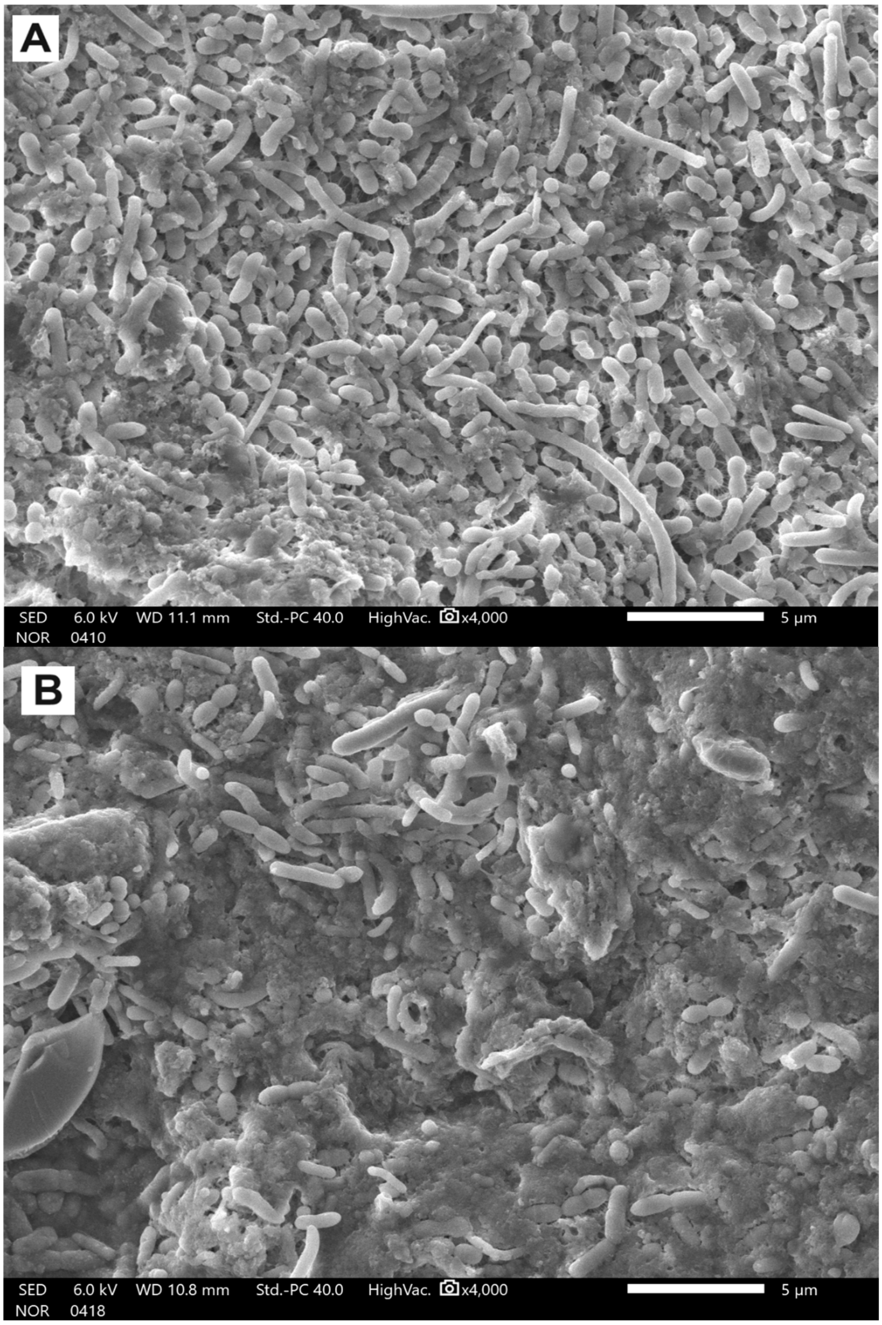

Figure S3. Imaging of a dry sludge sample at a critical point by scanning electron microscopy (SEM). (A) Sludge generated in untreated effluent; (B) sludge generated in effluent treated with guarana agroindustrial waste extract (500 mg/L).

2.1. Antibacterial Activity of Guarana Seed Peel Extract In Vitro (GSPE)

The LIVE/DEAD® BacLight™ Bacterial Viability Kit was essential to determine what is happening with microorganisms during the wastewater treatment with guarana extract. This kit offers an advantage over agar plate counting: cell death can be observed directly and in near real time, without an incubation period.

The method allows the detection of cellular states other than alive—dead cells and living injured cells that are unable to grow on agar plates. This fact increases the accuracy of analysis, because culture-based viability assessment can result in the underestimation of viable cells, which may affect potential applications. Finally, the Live/Dead staining allows membrane loss of integrity to be visualized over time [46].

2.2. Antibacterial Activity of Guarana Seed Peel Extract In Vitro

Cell viability was assessed with the LIVE/DEAD® BacLight™ Bacterial Viability Kit (Thermo Fisher). This kit includes the SYTO 9—a dye that stains green on a fluorescence microscope in a live bacterial cell—and another dye, propidium iodide (PI) stains red in case of a dead bacterial cell. The method for fluorescence analyses was followed as per the guidelines provided by the viability kit supplier. The images were obtained using a confocal microscope (Leica TCS SP8). Escherichia coli ATCC 25922 was grown in nutrient broth, and 25 mL of log-phase culture was used in each assay [32]. During the experiment, part of the samples was incubated with the guarana organic extract and part was not (positive control). Apart from that, a part of the samples was treated with a standard antibiotic amoxicillin and with the solvents used in the extraction (negative control). All tests were made in triplicate.

After the bacterial treatment with GSPE (Figure S4), it was possible to observe a large number of cells emitting propidium iodide fluorescence, as indicated by the increasing number of red stained cells during the treatment, resulting in the cell membrane's rupture. In addition to fully stained dead cells, several cells emitting green SYTO 9 fluorescence also showed red fluorescence in the cytoplasm, indicating that their membranes were already partially compromised.

Flavonoids exhibit potent antimicrobial properties through diverse mechanisms. Flavonoids disrupt bacterial cell integrity by increasing membrane permeability and altering cell morphology. They also suppress key genes involved in adhesion and biofilm formation, such as fnbA, fnbB, icaA, and clfA. Additionally, these compounds interfere with bacterial communication systems, including the agr system and LuxS/AI-2 pathway, and inhibit sortase A (SrtA)—a critical enzyme for surface protein anchoring [38]. Catechins, a subclass of flavonoids, induce endogenous oxidative stress in bacteria by increasing ROS levels, thereby reducing their antioxidant capacity, and disrupt biofilm formation by blocking amyloid fibre formation and the σ(E)-mediated outer membrane stress response. Furthermore, catechins target essential bacterial systems, such as the thioredoxin (Trx) and glutathione/glutaredoxin (Grx) systems, and specifically inhibit DXP activity—a key enzyme in the MEP biosynthetic pathway. Together, these mechanisms underscore the multitarget nature of catechins and flavonoids in combating bacterial infections [38,47,48].

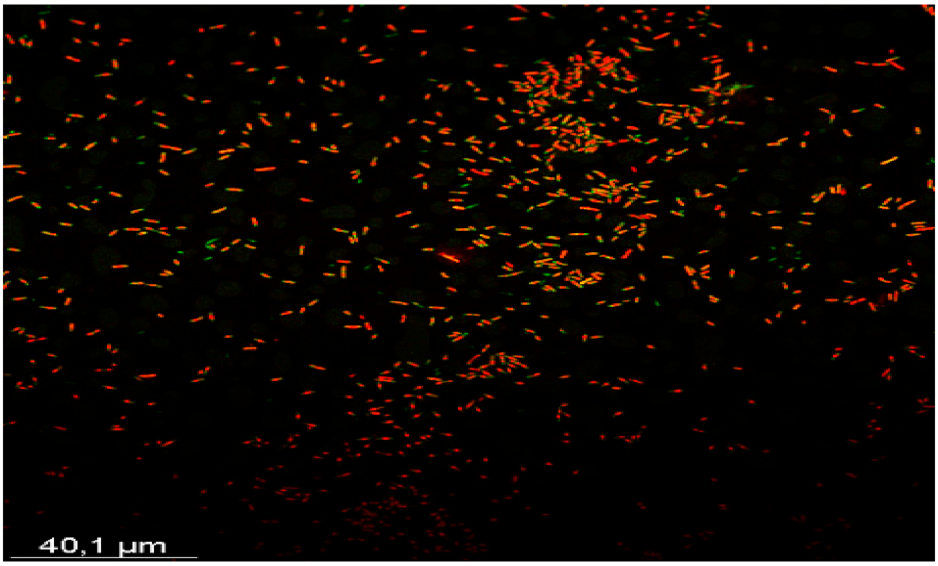

Figure S4. Imaging of the bacterial viability test in the samples treated with guarana agroindustrial waste extract using confocal microscopy.

In the Kirby–Bauer assay, GSPE produced no inhibition zone at any concentration tested, so no inhibitory concentration could be determined against E. coli. Another important fact to consider is that the antibacterial activity of plant extracts depends on the concentration used and the time of exposure to the products [4]. We attribute this to the crude nature of GSPE: because the active catechins are diluted among many co-extracted compounds, the amount loaded onto a disc was likely below the effective threshold (Figure S5). This result motivated the purification step, and the assay was repeated with the purified extract (GSPP)

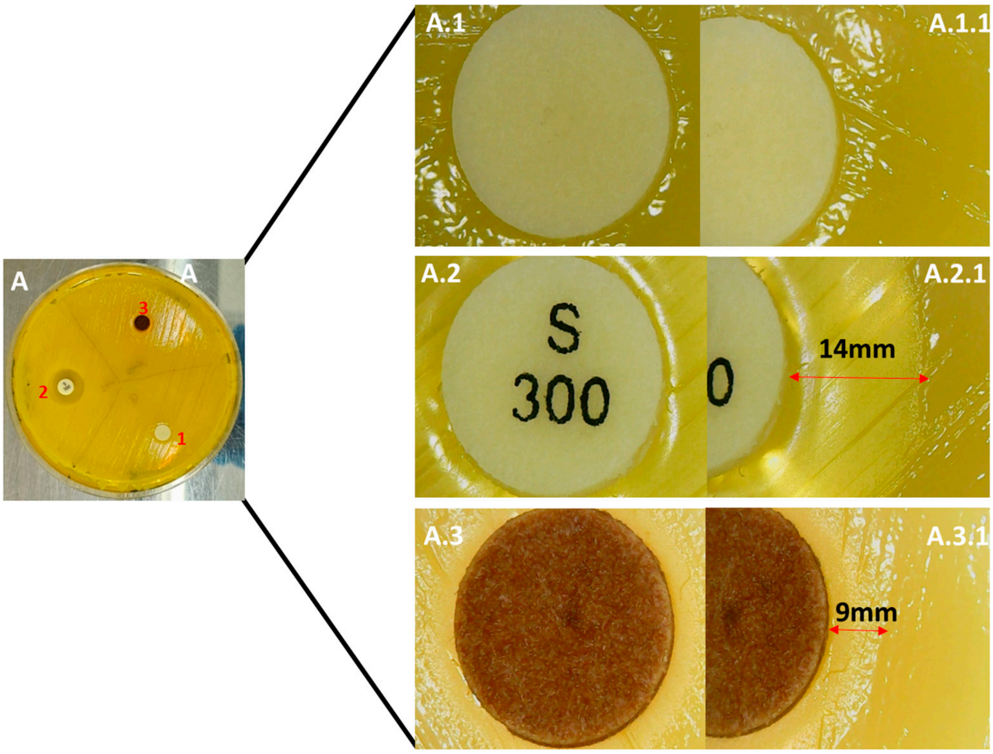

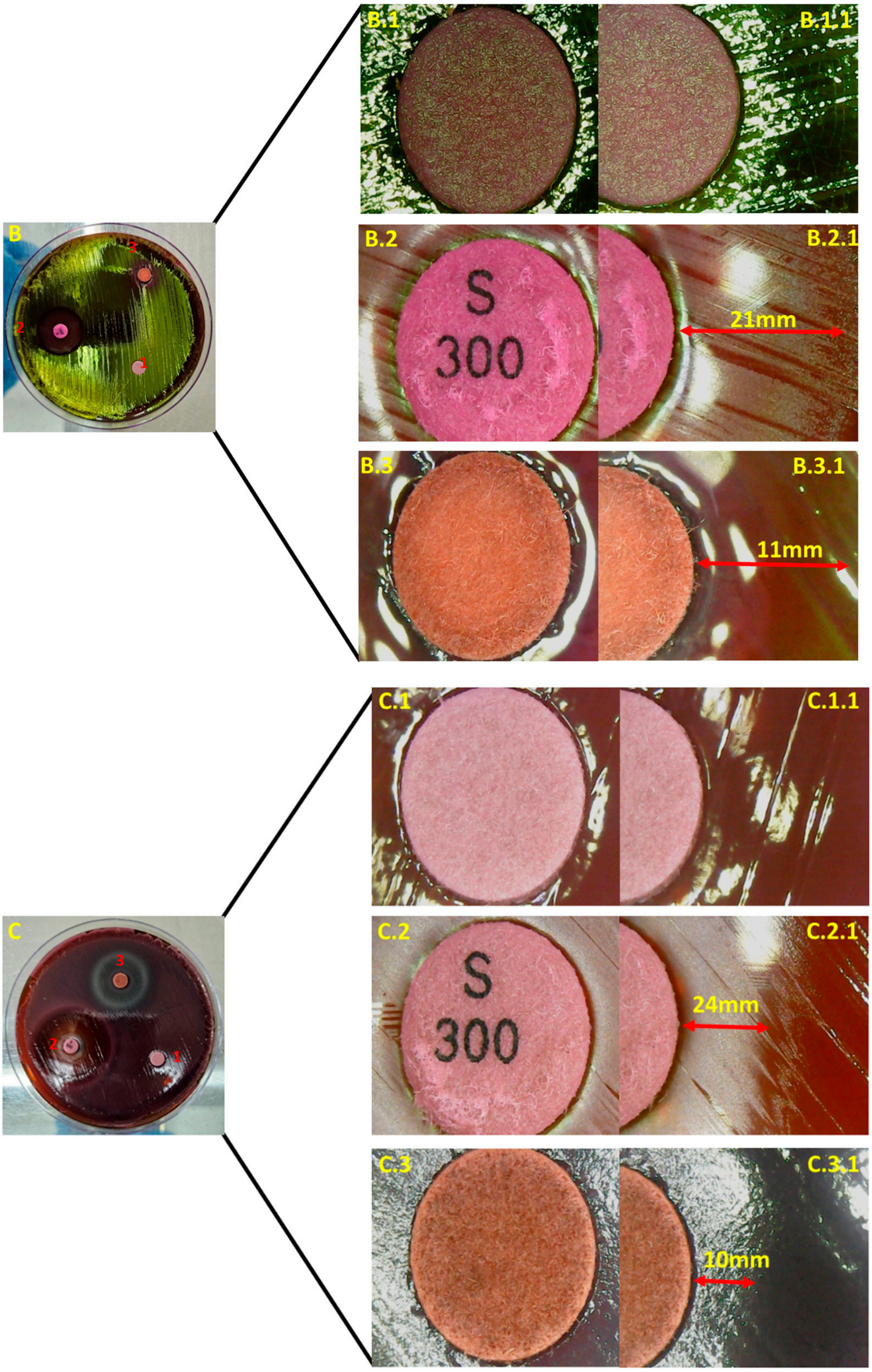

Figure S5. Disc diffusion assay with the purified guarana seed peel extract (GSPP) after incubation for 24 h at 37 °C. (A) Staphylococcus aureus ATCC 25923 on mannitol salt agar. (B) Escherichia coli ATCC 25922 on EMB agar. (C) Salmonella Typhimurium ATCC 14028 on EMB agar. Within each panel: A.1, B.1, C.1 — blank disc loaded with the diluent alone (water:acetone, 3:1, v/v); A.2, B.2, C.2 — disc loaded with streptomycin S (300 µg); A.3, B.3, C.3 — disc loaded with GSPP (5 mg). The paired magnified views (A.1.1–C.3.1) show the inhibition zones from which the diameters reported in Table 2 were measured.
